# Creatine for Cognition in Autism Spectrum Disorder: A novel therapeutic intervention?

**DOI:** 10.1192/j.eurpsy.2026.11251

**Published:** 2026-07-31

**Authors:** G. Singh, J. Ahuja, S. Gunturu, E. Hollander, S. Prasad, S. Fekrat, B. Sharifi

**Affiliations:** 1 Psychiatry, Albert Einstein College of Medicine, Bronx; 2 Mass Gen- Psychiatry , Harvard Medical School, Boston; 3 Psychiatry, Bronx Care, Bronx, United States; 4 Kabul University of Medical Sciences, Kabul, Afghanistan; 5 Citrus Health, Miami, United States

## Abstract

**Introduction:**

Cognitive impairments are common but often neglected in Autism Spectrum Disorder (ASD). These deficits are particularly evident in domains such as working memory, processing speed, and short-term memory. Current pharmacological and behavioral interventions provide only limited improvements in cognition. Emerging research suggests that disrupted brain energy metabolism, including mitochondrial dysfunction and abnormalities in creatine-phosphocreatine systems, may contribute to these impairments. Creatine is a naturally occurring compound that supports ATP regeneration in high-demand brain regions and has shown cognitive benefits in other populations.

**Objectives:**

This review aimed to evaluate the rationale for using creatine as a cognitive enhancer in ASD by reviewing literature related to brain energy metabolism, creatine’s neurological functions, and cognitive outcomes in both clinical and preclinical studies.

**Methods:**

We conducted a review of literature using PubMed, Google Scholar, and Scopus. Search terms included combinations of “creatine,” “cognition,” “autism,” “ASD,” “mitochondrial dysfunction,” “brain energy,” “working memory,” and “processing speed.” We selected peer-reviewed publications from the past two decades, including preclinical studies, clinical trials, case reports, and mechanistic reviews relevant to creatine and neuroenergetics in ASD.

**Results:**

Multiple studies report that individuals with ASD show signs of mitochondrial dysfunction, altered oxidative stress responses, and reduced cerebral creatine levels. Preclinical models of creatine transporter deficiency replicate several features of ASD and demonstrate cognitive rescue with creatine analogs. In human populations, creatine supplementation has been associated with modest improvements in memory, attention, and processing speed. These effects are especially relevant to the cognitive domains most commonly affected in ASD.

**Conclusions:**

Creatine supplementation may represent a low-risk and mechanistically plausible intervention for improving cognition in ASD. Based on the convergence of preclinical and translational evidence, we propose that future research should explore creatine as a cognitive enhancer in ASD, particularly in individuals with evidence of energy metabolism abnormalities. This review provides a foundation to guide the design of pilot studies targeting this promising yet understudied therapeutic pathway.

**Disclosure of Interest:**

None Declared

